# Overexpression of the Survivin gene in SGC7901 cell resistance to cisplatin

**DOI:** 10.3892/ol.2014.2463

**Published:** 2014-08-20

**Authors:** HANZHANG DONG, GAOGAO LIU, BIAO JIANG, JIUBING GUO, GUOQUAN TAO, WEI YIU, JINGSONG ZHOU, GUOXIN LI

**Affiliations:** 1 Department of General Surgery, The Affiliated Nanfang Hospital of Southern Medical University, Guangzhou, Guangdong 510515, P.R. China; 2 Department of General Surgery, The Affiliated Beijiao Hospital of Southern Medical University, Shunde, Guangdong 528311, P.R. China

**Keywords:** gastric cancer, chemotherapy, cisplatin, Survivin

## Abstract

The aim of the present study was to investigate the mechanism of SGC7901 cell resistance to cisplatin (CDDP). SGC7901/CDDP cells were established by the long-term continuous exposure of SGC7901 cells to CDDP in stepwise concentration increments. The morphologies of the SGC7901/CDDP and SGC7901 cells were observed by microscopy. The expression levels of Survivin mRNA and protein in the SGC7901/CDDP and SGC7901 cells were examined by reverse transcription polymerase chain reaction and western blotting respectively. The results revealed morphological differences between the SGC7901 and SGC7901/CDDP cells. The expression levels of Survivin mRNA and protein were significantly higher in the SGC7901/CDDP cells than in the SGC7901 cells. Therefore, high expression levels of the Survivin gene may explain SGC7901 cell resistance to CDDP.

## Introduction

Cisplatin (CDDP) is one of the most commonly used drugs in chemotherapy for gastric cancer. However, when gastric cancer cells develop resistance to CDDP, the chemotherapeutic effect of CDDP is reduced and this may even result in the failure of chemotherapy. Therefore, the analysis of CDDP resistance in gastric cancer cells has important implications. The Survivin gene is a member of the inhibitor of apoptosis protein family (1). Since Survivin inhibits apoptosis and is beneficial to the growth of tumor cells, the Survivin gene is also known as ‘survival factor’ (2). The Survivin gene is overexpressed in gastric cancer cells (3), which may be associated with the resistance of CDDP to gastric cancer cells and this was investigated in the present study.

## Materials and methods

### SGC7901 cell culture

Human gastric cancer SGC7901 cells were obtained from the cell bank of the Chinese Academy of Sciences (Shanghai, China). The SGC7901 cells were cultured in a humidified atmosphere of 5% CO_2_ and 95% air using RPMI 1640 (Invitrogen Life Technologies, Carlsbad, CA, USA) supplemented with 10% fetal bovine serum, 100 U/ml penicillin and 100 μg/ml streptomycin (both North China Pharmaceutical Group Corporation, Shijiazhuang, China) in 75-cm^2^ flasks at 37°C. The RPMI 1640 was adjusted to pH 7.2 with 5.6% sterile NaHCO_3_. The cell culture medium was changed every 2–3 days. The cells were subcultured when 80% confluence was reached.

### SGC7901/CDDP cell culture

RPMI 1640 with 100 ng/ml CDDP (lyophilized type; batch number, 6040122DC; Qilu Pharmaceutical Co., Ltd., Jinan, China) was added to the culture medium and the medium was changed every 2–3 days. When 80% confluence was reached, the cells were subcultured with RPMI 1640 to achieve a good adhesive condition. As the cells became adherent to the bottom of cell culture flasks, RPMI 1640 with 200 ng/ml CDDP was added to the medium and the medium was changed every 2–3 days. When the cells has reached 80% confluence, the cells were subcultured with RPMI 1640 to achieve a good adhesive condition. As the cells became adherent to the bottom of the cell culture flasks, RPMI 1640 with 500, 700 or 1,000 ng/ml CDDP was added to the medium and the medium was changed every two-three days.

### Survivin mRNA detection

Total RNA was extracted from the cells using TRIzol reagent (Invitrogen Life Technologies) and cDNA synthesized from RNA (1 μg) was used as a template for the RT reaction (Invitrogen Life Technologies). The 447-bp Survivin DNA fragment was amplified using the following two primers synthesized by Invitrogen Life Technologies: Forward, 5′-GCATGGGTGCCCCGACGTTG-3′ and reverse, 5′-GCTCCGGCCAGAGGCCTCAA-3′. Polymerase chain reaction (PCR) was performed in a solution containing 2 μl 10X PCR buffer (Invitrogen Life Technologies), 0.8 μl MgCl_2_, 1.0 μl dNTPs (Invitrogen Life Technologies), 0.2 μl of each primer, 2.0 μl cDNA and 1.0 μl Taq DNA polymerase (Promega Corporation, Madison, WI, USA), to obtain a total volume of 20 μl. The amplification was performed in a microcentrifuge tube under the following conditions: Denaturation at 94°C for 30 sec, annealing at 55°C for 60 sec and elongation at 72°C for 60 sec, for 30 cycles. The 241-bp β-actin fragment was amplified using the following two primers synthesized by Invitrogen Life Technologies: Forward, 5′-TAAAGACCTCTATGCCAACACAGT-3′ and reverse, 5′-CACCATGGAGGGGCCGGACTCTTC-3′. PCR was performed in a solution containing 2 μl 10X PCR buffer (Invitrogen Life Technologies), 1.6 μl MgCl_2_, 1.0 μl dNTPs (Invitrogen Life Technologies), 0.2 μl of each primer, 2.0 μl cDNA and 1.0 μl Taq DNA polymerase (Promega Corporation) to obtain a total volume of 20 μl. The amplification conditions were as follows: Denaturation at 94°C for 30 sec, annealing at 58°C for 40 sec and elongation at 72°C for 40 sec, for 28 cycles. The PCR products were separated on a 1% agarose gel containing ethidium bromide. The gel images were digitally recorded and analyzed by computer assisted image analyzer with Lab-work 4.5 analysis software (Ultra Violet Products, Upland, CA, USA). The relative content of Survivin mRNA was indicated by the absorbance ratio of the Survivin mRNA band and the β-actin band.

### Survivin protein detection

The SGC7901 and SGC7901/CDDP cells were washed twice with 4°C phosphate-buffered saline (PBS). RIPA buffer (Invitrogen Life Technologies) was added and the cells were lysed on ice for 30 min, then clarified by centrifugation at 10,000 × g for 10 min at 4°C. The supernatants were used to assay protein concentrations. Subsequently, 25 μg protein was loaded and separated by polyacrylamide gel electrophoresis and then transferred to a polyvinylidene difluoride (PVDF) membrane. The PVDF membranes were incubated for 2 h at room temperature with 5% skimmed powdered milk in 500 mm NaCl, 20 mm Tris-HCL (pH 7.5) and 0.5% PBS-Tween 20, and then for 24 h at 4°C with the following dilutions of primary antibody: 1:2,000 anti-human Survivin antibody (catalog number, AF6471; immunoglobulin-type, human Survivin specific goat IgG; R&D Systems, Minneapolis, MN, USA) and 1:500 anti-β-actin antibody (Wuhan Boster Biological Technology, Ltd., Wuhan, China). Subsequent to being washed with Tris-buffered saline-Tween 20, the PVDF membranes were incubated with 1:3,000 peroxidase-conjugated rabbit anti-goat secondary antibodies (Wuhan Boster Biological Technology, Ltd.) for 2 h at room temperature. Proteins were visualized using chemiluminescent peroxidase substrate (Pierce Biotechnology, Inc., Rockford, USA), and the protein blots were quantified and analyzed by computer-assisted image analyzer with Lab-work 4.5 analysis software. The relative content of Survivin protein was indicated by the absorbance ratio of the Survivin protein band to the β-actin band.

### Statistical analysis

Data are expressed as the mean ± standard deviation. Student’s t-test was used for comparisons involving two groups. All statistically analyses were performed using SPSS 19.0 software (IBM, Armonk, NY, USA). P<0.05 was considered to indicate a statistically significant difference.

## Results

### Morphology of SGC7901 and SGC7901/CDDP cells

**Figure 1:**
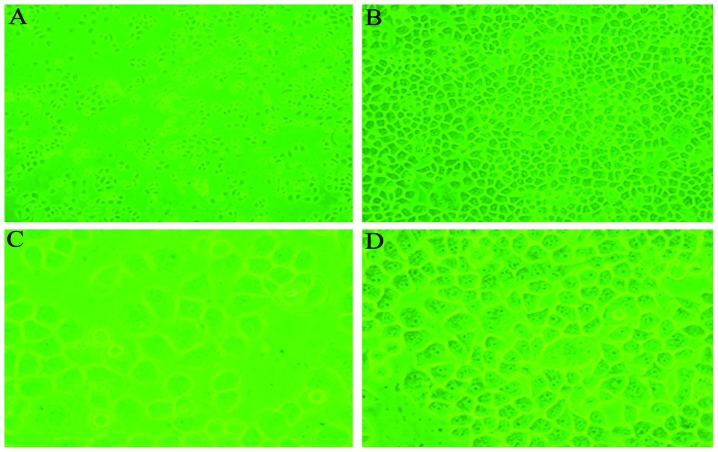
SGC7901 and SGC7901/CDDP human gastric cancer cell morphology under light microscopy. (A) SGC7901/CDDP cells (magnification, ×100).(B) SGC7901 cells (magnification, ×100). (C) SGC7901/CDDP cells (magnification, ×400). (D) SGC7901 cells (magnification, ×400). CDDP, cisplatin.

The SGC7901 cells appeared polygonal, cobblestone-like and tightly adherent to the flask, and were highly refractive and proliferative. The SGC7901/CDDP cells were relatively reduced in number, only marginally refractive and weakly adherent to the flask, and increased space was observed between the cells. A few SGC7901/CDDP cells were deformed, increased in size or floating in the culture medium (Fig. 1).

### Survivin mRNA and protein expression levels in SGC7901 and SGC7901/CDDP cells

**Figure 2:**
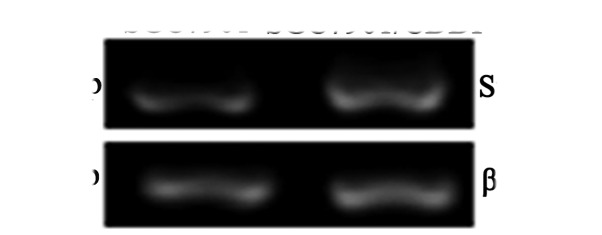
Survivin mRNA expression levels in SGC7901 and SGC7901/CDDP human gastric cancer cells examined by reverse transcription polymerase chain reaction analysis. CDDP, cisplatin.

**Figure 3:**
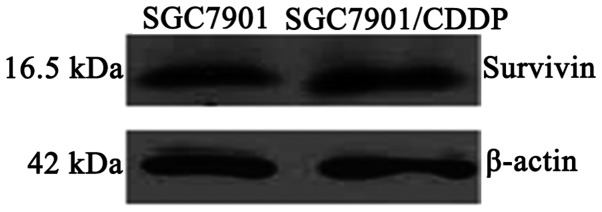
Survivin protein expression levels in SGC7901 and SGC7901/CDDP human gastric cancer cells examined by western blot analysis. CDDP, cisplatin.

Survivin mRNA expression levels were significantly higher in the SGC7901/CDDP cells compared with the SGC7901 cells (P<0.05; Table I; Fig. 2). Survivin protein expression levels were also significantly higher in the SGC7901/CDDP cells compared with the SGC7901 cells (P<0.05; Table I; Fig. 3).

## Discussion

Gastric cancer is a malignant disease with high morbidity and mortality. Since gastrointestinal tumors are sensitive to chemotherapy, this form of therapy is an indispensable element in the comprehensive treatment of gastric cancer. In recent years adjuvant chemotherapy and neoadjuvant chemotherapy have been increasingly used in clinical practice, but the survival times of patients with gastric cancer have not been significantly prolonged (3). One reason for this is the resistance of gastric cancer cells to chemotherapeutic drugs. CDDP remains a commonly used classical drug for patients with gastric cancer. CDDP kills tumor cells by binding the DNA, forming cross-links with the DNA, and subsequently inhibiting DNA synthesis and the division of the cancer cells (4–6). In tumor cell resistance to CDDP, the efficacy of CDDP is reduced, even resulting in the failure of chemotherapy in patients with gastric cancer. However, the mechanism of chemotherapy resistance of gastric cancer cells to CDDP is unclear.

To clarify the underlying mechanism of the chemotherapeutic resistance of gastric cancer cells to CDDP, a CDDP-resistant gastric cancer cell strain was established *in vitro*. At present, there are three methods to establish resistant tumor cell strains, consisting of the stepwise exposure of cells to increasing concentrations of the drug (7–9), low doses of the drug (intermittent induced method) (10) and high doses of the drug (intermittent induced method) (11). In the present study, SGC7901/CDDP cells were established by the stepwise exposure of SGC7901 cells to increasing CDDP concentrations.

Goldie and Coldman (12) considered there to be two types of drug resistance in tumor cells, namely endogenous drug resistance and acquired drug resistance. Acquired drug resistance in tumor cells indicates that the sensitivity of tumor cells to drugs is progressively reduced, thus the drug becomes less effective or even ineffective; a mechanism similar to the development of antibiotic resistance. Conversely, endogenous drug resistance does not undergo a gradual desensitization process; the resistance is already present prior to the initiation of drug treatment. The SGC7901/CDDP strain produced in the present study underwent the former process. Drug resistance may involve the mutation of tumor cells during cell growth and proliferation. Resistant cells are produced in each mutation. The cells that adapt to the mutation during this time may have a novel mutation which has been induced by the change in drug concentrations. Thus, drug resistance is not an all-or-nothing phenomenon, but a gradual process (13). The establishment of the SGC7901/CDDP cells was also a gradual process in the present study. As the drug concentration was continuously increased in the culture medium, the CDDP resistance strain, SGC7901/CDDP, was developed.

The established SGC7901/CDDP cells exhibited stable growth and proliferation following cryopreservation, and long-term culture indicated that the resistance of the cells to CDDP was relatively stable. Therefore, the SGC7901/CDDP cell line was reliable and an ideal cell model for analyzing the mechanism of CDDP resistance. The majority of drug-resistant strains exhibit multidrug-resistant characteristics. Whether the SGC7901/CDDP cells are resistant to other commonly used chemotherapy drugs, such as 5-fluorouracil, paclitaxel and hydroxycampothecin, requires further investigation.

Comparing the morphology of SGC7901 and SGC7901/CDDP cells by microscopy, the SGC7901/CDDP cells were reduced in number, with certain cells deformed or increased in size. Giant cells were also visualized, demonstrating that the cells were damaged. The degree of refraction had declined, the space between the cells was increased, the cells were less adherent to the flask and a few cells were floating in the culture medium. Reduced cell proliferation was also observed. Survivin mRNA and protein were expressed in the SGC7901 and SGC7901/CDDP cells, but the expression levels were significantly higher in the SGC7901/CDDP cells compared with the SGC7901 cells. The results suggest that the induction of increased Survivin gene expression levels is a cause of CDDP resistance in gastric cancer cells.

One study has shown that the knockdown of Survivin expression enhances the sensitivity of gastric cancer cells to CDDP (12). Furthermore, overexpression of the Survivin gene in gastric cancer cells has been associated with the resistance to docetaxel-based chemotherapy in patients with advanced gastric cancer (15), and the overexpression of the Survivin gene induced by CDDP has been demonstrated to aid cancer cells in overcoming the apoptosis checkpoint at the G_2_/M phase of the cell cycle (16). Prior to chemotherapy, the analysis of Survivin gene expression does not indicate whether the tumor is sensitive or resistant to chemotherapeutic drugs. However, during chemotherapy, the assessment of Survivin gene expression may provide novel information regarding tumor drug sensitivity (17), although the mechanism is unclear (18). Therefore, Survivin gene expression levels may add significant prognostic value to the current tumor-node-metastasis staging system (19), and the correlation between the expression of Survivin and overall survival time for patients with gastric cancer is evident (20).

In the intraocular environment of the body, the metabolic changes and drug resistance induced by CDDP are difficult to explain clearly, and the underlying mechanism may not be elucidated simply by establishing a cell model *in vitro*. Thus, the underlying mechanism with regard to the development of chemotherapeutic resistance to CDDP in gastric cancer cells requires further investigation.

## Figures and Tables

**Table I:** Survivin mRNA and protein expression levels in SGC7901 and SGC7901/CDDP human gastric cancer cells.

	Cell type		
		
Parameter	SGC7901	SGC7901/CDDP	P-value
mRNA	0.748±0.011	1.010±0.068	<0.05
Protein	1.430±0.234	2.565±0.382	<0.05

CDDP, cisplatin.
